# Early effect of NEURAPAS^® ^balance on current source density (CSD) of human EEG

**DOI:** 10.1186/1471-244X-11-123

**Published:** 2011-08-02

**Authors:** Wilfried Dimpfel, Klaus Koch, Gabriele Weiss

**Affiliations:** 1 Justus-Liebig-University c/o NeuroCode AG, Sportparkstr. 9, 35578 Wetzlar, Germany; 2 NeuroCode AG, Sportparkstr. 9, 35568 Wetzlar, Germany; 3 Pascoe Pharmazeutische Präparate GmbH, 35394 Giessen, Germany

## Abstract

Psychiatric patients often suffer from stress, anxiety and depression. Various plant extracts are known to fight stress (valerian), anxiety (passion flower) or depression (St. John's wort). NEURAPAS^® ^balance is a mixture of these three extracts and has been designed to cover this complex of psychiatric conditions. The study was initiated to quantitatively assess the effect of this combination on brain electric activity.

**Method:**

Quantitative electroencephalogram (EEG) current source density (CSD) recording from 16 healthy male and female human volunteers (average age 49 years) was used in a randomized, placebo-controlled cross over study. Recordings were performed 0. 5, 1. 5, 3 and 4 hours after administration of the preparations under the conditions of 6 min eyes open and 5 min d2 concentration test, mathematical calculation test and memory test, respectively. All variables (electric power within 6 frequency ranges at 17 electrode positions) were fed into a linear discriminant analysis (eyes open condition). In the presence of mental load these variables were used to construct brain maps of frequency changes.

**Results:**

Under the condition of mental load, centro-parietal spectral power remained statistically significantly lower within alpha1, alpha2 and beta1 frequencies in the presence of verum in comparison to placebo. Discriminant analysis revealed a difference to placebo 3 and 4 hours after intake of 6 tablets of NEURAPAS^® ^balance. Data location within the polydimensional space was projected into the area of the effects of sedative and anti-depressive reference drugs tested earlier under identical conditions. Results appeared closer to the effects of fluoxetine than to St. John's wort.

**Conclusions:**

Analysis of the neurophysiological changes following the intake of NEURAPAS^® ^balance revealed a similarity of frequency changes to those of calming and anti-depressive drugs on the EEG without impairment of cognition.

**Trial registration:**

ClinicalTrials.gov: NCT01047605

## Background

People today face quite a number of psychic challenges. Working life produces numerous stress situations, which sometimes are difficult to cope with. Permanent pressure of this kind in many cases leads to adaptive mechanisms of the brain and successful coping. But there are also numerous cases where these protective mechanisms do not work or show exhaustion with time. Very often anxiety and depression are the result of such continuous environmental exposures. Under theses circumstances patients need help. Broad use of synthetic drugs like benzodiazepines or serotonin reuptake inhibitors is for example accompanied by serious side effects like cognitive impairment in the case of benzodiazepines [1] or heart and circulation problems in the case of reuptake inhibitors [2]. An alternative solution can be provided by administration of natural remedies. For centuries the calming effect of passion flower and valerian root extract have been recognized and were partially confirmed in recent clinical studies. For example preoperative oral Passiflora incarnate reduces anxiety in ambulatory surgery patients [3] and there is tentative evidence that extract of Valeriana wallichii attenuated stress and anxiety [4]. Efficacy and tolerability of Hypericum extract (St. John's wort) for the treatment of mild to moderate depression has been documented [5]. This knowledge has resulted in numerous formulations of dietary supplements and herbal extracts for relaxation and anxiolytic action (for review s. [6]. Nearly, all clinical studies dealing with the treatment of depression by St. John's wort have provided solid evidence for its efficacy during treatment. But with respect to anxiety there is a lack of rigorous studies in this area [7].

In order to cover a broader range of possible psychiatric distortions a film-coated tablet with a mixture of extracts of valerian root, passion flower herb and St. John's wort herb has been developed for pharmacological treatment of these conditions under the name of NEURAPAS^® ^balance. This combination of extracts has been characterized pharmacologically in vitro and in vivo [8]. It was shown for the first time that Passiflora significantly enhances the potency of Hypericum (St. John's wort) in two preclinical models: serotonin reuptake and forced swimming test.

With the availability of new encephalographic techniques like continuous online current source density measurements (CATEEM^®^) the quantitative description of brain function has entered a stage where the measurement of even very subtle changes of physiological brain activity has become feasible [9]. The aim of the present clinical study was to substantiate the effectiveness of this preparation by current source density (CSD) analysis of brain activity in the presence of various mental challenges. This technology-as a special form of quantitative pharmaco-EEG-has been used widely in the past for the characterization of drug effects.

## Methods

### Subjects

Sixteen healthy volunteers (8 males, 48,7 ± 7 years old and 8 females, 47 ± 8 years old) were recruited by advertisement and participated in this study. They underwent a medical examination before entering the trial. Subjects reporting neurological disturbances of the central nervous system (using DSM-III criteria) were excluded from the study. Subjects with a history of drug or ethanol abuse or participation in another study within the last six months were also excluded. It was ensured that they were not on alcohol. On the day of examination no beverages containing caffeine were allowed within the last 12 hours preceding the EEG recording. The study was carried out according to the declaration of Helsinki [10] on human rights and was approved by the local ethics committee of the State Medical Association Hessen (Frankfurt, Germany) and government authorities (BfArM, Bonn, Germany). All subjects were informed about the goals of the study in detail and gave their written informed consent to participate. Each subject was randomly allocated to either the functionally active preparation or placebo within a crossover design.

### EEG Recording

Subjects sat alone in a quiet separate room in a comfortable easy chair. The light was dimmed. Baseline recording of 6 min under the condition of eyes open was followed by performance of three cognitive tests: a concentration test (d2-test) [11], a mathematical calculation test (according to [12]) and a memory test consisting in the presentation of a row of letters and numbers, which appeared for 4 s on a screen followed by 10 s black screen. After this a multiple choice of four possible answers was presented.

**Figure 1:**
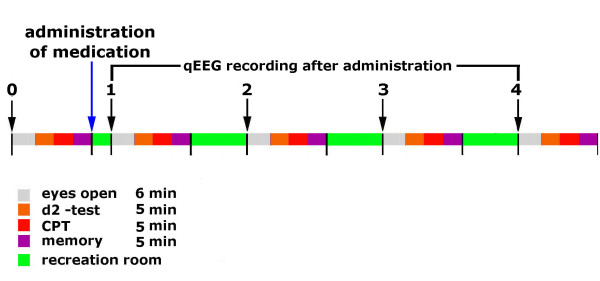
**Time line of EEG recording**. Time line of EEG recording before and after administration of the preparations.

All recordings were repeated 0. 5 h, 1. 5 h, 3 and 4 h after the administration of the tablets (overview in Figure 1). Between the measurement sessions the subjects spent their time in the facility's break room. All experiments took place at the same time of the day (starting at 8 h a. m.).

The EEG was recorded bipolarly from 17 surface electrodes according to the international 10/20 system with Cz as physical reference electrode for calculation of the common average reference (Computer aided topographical electroencephalometry: CATEEM^® ^- from Mewicon GmbH, 4164 Schwarzenberg, Austria) using an electrocap [13]. The raw signals were amplified, digitalized (2048 Hz/12 bit) and transmitted via fiber optical devices to the computer. The automatic artefact rejection of the CATEEM^®^-System, which eradicates EEG-alterations caused by eye blinks, swallows, respiration etc. during the recording was visually controlled and individually adjusted by the investigator. Electrocardiogram (ECG) and electrooculogram (EOG) were recorded in one channel each in order to facilitate detection of those signals superposed on to the EEG. The artefact rejection set-up was observed for about 5 minutes prior to the start of the recording to ensure, that all artefacts were correctly recognized and eliminated from further evaluation. For safety purposes the original raw data were saved on optical disk in order to allow re-evaluation of the artefact rejection mode if necessary. In these cases the experimental session was re-examined off-line with a newly adapted rejection mode. The amount of rejected data was determined automatically and given in percent of total recording time. Nevertheless the entire recording and the computer-based automatic artefact rejection were continuously supervised and adjusted by a trained technician [14]. Logarithm of CSD data was taken for discriminant analysis in order to approach normal distribution of values, which underwent frequency analysis for quantitative evaluation as proposed for the first time nearly 80 years ago [15].

In this study the EEG was computed not in the potential mode measured as voltage, but in a surface charge mode obtained by Laplacian estimates also known as current source density (CSD) analysis [16]. The charge is the 2^nd ^derivative of the potential and gives the spatial curvature of the potential. All calculations are based on the standard set-up of the 10/20 system of recording. Under the condition of using a homogenous, steadily conducting medium the surface charge mode provides the source density of the electrical flow on the cortex surface. Whereas the EEG in the potential mode tends to produce a more extensive and diffuse picture of changes, the Laplacian estimate acts as a spatial filter emphasizing local sources over distant sources. Others [16] were able to demonstrate, that spectral parameters obtained from the CSD showed higher correlations with computer tomography measures than those calculated from the potential mode of the EEG. We therefore used this methodology in order to describe the focal changes of brain activity. Using a Lagrange interpolation, signals from 82 additional virtual electrodes were calculated to provide high-resolution topographical maps. The signals of all 99 electrode positions (17 real and 82 virtual) underwent the Fast Fourier Transformation (FFT) based on 4-second sweeps of data epochs (Hanning window). Data were analysed from 0. 86 to 35 Hz using the CATEEM^® ^software. In this software the resulting frequency spectra are divided into six frequency bands: delta (1. 25-4. 50 Hz), theta (4. 75-6. 75 Hz), alpha1 (7. 00-9. 50 Hz), alpha2 (9. 75-12. 50 Hz), beta1 (12. 75-18. 50 Hz) and beta2 (18. 75-35. 00 Hz). This frequency analysis is based on absolute spectral power values. Colour coding of the maps is achieved by transforming the content of the power spectrum into the so-called colour vision mode pictures (i. e. frequencies are converted into spectral colours with a resolution of 0. 25 Hz, which gives 140 colours which are then mixed according to spectral content to give one map similar to the RGB mode of TV pictures). Data acquisition and analysis were carried out simultaneously and provide topographical maps displayed on-line on the computer screen. The maps show the relative, time-averaged, functional changes of electric brain activity of each specific recording condition in comparison to the reference period at the beginning of each recording session during the relaxed state with open eyes.

Band powers are firstly compared between placebo and verum during relaxation, then the effects of different mental loads are determined in the absence of either placebo or verum, and finally the results of verum and placebo are compared under different mental loads.

### Statistics

EEG data from the first recording session before drug intake are given as absolute numbers (μV^2^). By setting the absolute electric power of this first pre-drug recording to 100%, changes produced by the preparations are given as percentage from this pre-drug condition. Except for the construction of brain maps only those particular electrode positions were used for the numeric comparison of verum to placebo during mental load, which have been found to change during the particular mental challenge. For explorative statistical evaluation the nonparametric sign test was used. For mathematical classification of drug effects the linear discriminant analysis according to Fischer was used. Results from the first three discriminant functions were projected into space (x, y and z coordinates), whereas results from the fourth to sixth discriminant functions were coded into red, green and blue, respectively, followed by an additive colour mixture (so-called RGB-mode). Reference preparations used for comparison have been tested in our lab earlier under identical conditions. These data were processed by linear discriminant analysis. Thereafter we used the same transformation by using this projection without any further discriminant analysis procedure for depiction of Neurapas^® ^balance.

Psychometric performance was evaluated by the mathematical product of "quantity × quality", where quantity was defined as the number of correct answers and quality as the number of correct answers divided by the number of tasks tackled.

### Preparation

NEURAPAS^® ^balance is a film-coated tablet containing a combination of special dried extracts: 60 mg "St John's wort Herb" (4. 6-6. 5:1), extractant: ethanol 38% (m/m), 28 mg "Valerian root" (3. 8-5. 6:1), extractant: ethanol 40% (m/m), and 32 mg "passion flower herb" (6. 25-7. 1:1), extractant: ethanol 60% (m/m). NEURAPAS^® ^balance is registered in different European countries for the treatment of mild depression, low mood, anxiety, and sleep disorders and marketed by Pascoe Pharmazeutische Präparate GmbH, Giessen, Germany. The verum preparation consisted of the maximum daily dosage of 6 tablets of NEURAPAS^® ^balance. At least one week passed between administrations within the crossover design.

## Results

### Analysis of Single Frequency Ranges during Relaxation

Source density analysis of the primary recording of the reference EEG before drug administration revealed similar absolute power values for the first day of placebo administration (Pl) in comparison to the first day of active drug administration (Neu) for all electrode positions as documented in Table 1. Following drug administration no changes were documented between placebo and verum within the first 1. 5 hours (not shown). Three hours after administration lower spectral power values were seen in general for the verum condition (please compare median values in Table 2) compared to placebo values. Only one highly statistically significant value was recognized at the electrode position P4. At four hours after administration again lower values were obtained in general but only alpha waves-particularly alpha2 frequencies at the electrode positions C3, T3, P4 and O1- showed differences to placebo values (Table 3).

**Table 1:** Absolute spectral power values

		Delta		Theta		Alpha 1		Alpha 2		Beta 1		Beta 2	
**Table of absolute values eyes open 0 h**													

**E**	**n**	**Pl**	**Neu**	**Pl**	**Neu**	**Pl**	**Neu**	**Pl**	**Neu**	**Pl**	**Neu**	**Pl**	**Neu**

**Cz**	16	3. 35	4. 34	0. 60	0. 78	0. 55	0. 73	0. 43	0. 54	0. 67	0. 76	0. 90	1. 22

**Fz**	16	3. 42	2. 89	0. 68	0. 76	0. 67	0. 66	0. 66	0. 62	0. 54	0. 55	0. 82	0. 93

**F3**	16	2. 75	2. 45	0. 71	0. 59	0. 76	0. 76	0. 69	0. 54	1. 10	0. 65	2. 04	1. 60

**C3**	16	1. 67	2. 49	0. 46	0. 54	0. 67	0. 78	0. 59	0. 54	1. 17	1. 44	1. 75	2. 21

**P3**	16	1. 70	1. 71	0. 36	0. 40	0. 54	0. 42	0. 60	0. 47	0. 71	0. 80	0. 58	0. 67

**Pz**	16	1. 95	2. 92	0. 48	0. 55	0. 75	0. 76	0. 74	0. 70	0. 73	0. 84	0. 49	0. 60

**P4**	16	1. 63	1. 83	0. 35	0. 43	0. 61	0. 58	0. 63	0. 74	0. 63	0. 71	0. 46	0. 65

**C4**	16	1. 74	2. 23	0. 39	0. 49	0. 56	0. 74	0. 84	0. 73	1. 19	1. 38	1. 63	1. 52

**F4**	16	3. 22	2. 93	0. 64	0. 55	0. 67	0. 86	0. 61	0. 58	1. 07	0. 95	1. 75	2. 57

**F7**	16	10. 21	9. 86	1. 39	1. 42	1. 41	1. 52	1. 23	1. 40	2. 07	1. 67	3. 66	5. 09

**T3**	16	3. 22	5. 21	0. 77	0. 72	0. 83	1. 18	0. 97	1. 18	1. 56	2. 66	3. 25	5. 21

**T5**	16	3. 32	3. 13	0. 83	0. 83	1. 48	1. 68	1. 15	1. 27	1. 66	1. 89	1. 53	1. 66

**O1**	16	3. 41	4. 01	0. 60	0. 84	0. 86	1. 07	0. 81	0. 85	1. 22	1. 45	2. 18	1. 70

**O2**	16	3. 33	3. 87	0. 61	0. 75	0. 78	1. 21	1. 26	1. 17	1. 18	1. 85	1. 94	2. 47

**T6**	16	2. 38	3. 06	0. 67	0. 74	1. 44	1. 51	1. 26	1. 07	1. 24	1. 45	1. 33	1. 22

**T4**	16	4. 36	3. 84	0. 72	0. 70	0. 99	0. 81	1. 04	0. 76	1. 47	1. 31	4. 65	2. 61

**F8**	16	7. 09	10. 47	1. 30	1. 32	1. 45	1. 57	1. 27	1. 38	2. 03	2. 41	5. 27	5. 76

**Me**	16	3. 22	3. 06	0. 64	0. 72	0. 76	0. 81	0. 81	0. 74	1. 18	1. 38	1. 75	1. 66

Absolute spectral power values for each electrode position **before administration **of drug or placebo during relaxation under the recording condition of eyes open. Data are given in μV^2^. These data are taken as 100% reference for further evaluation. E = Electrode; Me = Median; n = number of subjects.

**Table 2:** Relative spectral power values 3 h after intake of placebo or verum

		Delta		Theta		Alpha 1		Alpha 2		Beta 1		Beta 2	
**Relative Change of Power (Eyes open) 3 h**													

**E**	**n**	**Pl**	**Neu**	**Pl**	**Neu**	**Pl**	**Neu**	**Pl**	**Neu**	**Pl**	**Neu**	**Pl**	**Neu**

**Cz**	16	76. 26	80. 86	141. 47	101. 74	167. 62	148. 57	143. 90	129. 48	138. 02	136. 95	117. 94	112. 48

**Fz**	16	89. 24	101. 39	113. 30	124. 66	131. 00	124. 75	115. 87	97. 27	130. 94	108. 74	120. 41	118. 65

**F3**	16	108. 84	95. 86	132. 14	138. 32	131. 94	135. 07	126. 42	133. 43	144. 76	157. 32	184. 04	137. 30

**C3**	16	142. 33	87. 68	143. 49	108. 64	169. 91	122. 99	152. 78	125. 20	147. 19	117. 70	106. 15	122. 51

**P3**	16	127. 85	98. 28	145. 54	126. 43	132. 27	136. 77	121. 91	127. 87	109. 91	120. 80	94. 47	103. 24

**Pz**	16	130. 78	78. 11	150. 01	111. 89	168. 78	164. 56	135. 02	151. 40	113. 80	116. 45	110. 13	116. 63

**P4**	16	164. 93	**85. 49**	139. 43	109. 61	138. 61	130. 38	135. 47	121. 61	120. 43	110. 64	120. 88	99. 43

**C4**	16	105. 90	95. 90	132. 31	106. 03	157. 52	140. 94	147. 72	132. 68	138. 18	116. 96	111. 74	103. 87

**F4**	16	94. 84	102. 06	127. 60	135. 14	168. 46	120. 06	153. 12	115. 03	152. 21	124. 18	151. 04	123. 12

**F7**	16	90. 67	130. 51	128. 58	144. 18	130. 13	135. 33	129. 85	152. 14	142. 09	129. 21	135. 91	148. 81

**T3**	16	138. 06	113. 38	159. 04	148. 86	194. 55	150. 60	152. 94	151. 82	124. 67	128. 47	89. 64	129. 67

**T5**	16	133. 63	112. 75	157. 72	123. 87	174. 70	149. 38	133. 22	118. 85	111. 39	123. 76	102. 13	100. 98

**O1**	16	115. 66	99. 28	131. 58	134. 39	144. 72	109. 64	132. 79	106. 45	144. 80	119. 48	128. 06	119. 81

**O2**	16	125. 27	87. 80	146. 25	112. 16	151. 53	104. 05	136. 12	118. 63	138. 64	112. 42	136. 18	105. 26

**T6**	16	147. 70	130. 73	154. 25	134. 24	156. 65	156. 08	145. 83	131. 83	119. 85	117. 06	94. 77	93. 16

**T4**	16	126. 46	134. 52	134. 62	136. 48	148. 01	160. 42	140. 19	133. 87	112. 93	111. 05	93. 34	109. 90

**F8**	16	138. 90	139. 66	140. 93	149. 02	129. 44	141. 96	130. 45	114. 66	125. 83	117. 55	126. 19	102. 40

**Me**	16	126. 46	99. 28	140. 93	126. 43	151. 53	136. 77	135. 47	127. 87	130. 94	117. 70	117. 94	112. 48

Relative power values (% of pre-drug) for each electrode position 3 h after administration of drug or placebo during relaxation under the recording condition of eyes open 4 h after intake of placebo or verum. Statistically significant different values in comparison to placebo (p = 0. 021) are printed **fat**. E = Electrode; Me = Median; n = number of subjects.

**Table 3:** Relative spectral power values 4 h after intake of placebo or verum

		Delta		Theta		Alpha 1		Alpha 2		Beta 1		Beta 2	
**Relative Change of Power (Eyes open) 4 h**													

**E**	**n**	**Pl**	**Neu**	**Pl**	**Neu**	**Pl**	**Neu**	**Pl**	**Neu**	**Pl**	**Neu**	**Pl**	**Neu**

**Cz**	16	92. 93	85. 06	120. 76	115. 00	160. 49	134. 92	178. 70	129. 99	134. 43	136. 66	129. 63	119. 80

**Fz**	16	110. 12	106. 59	126. 85	126. 92	153. 09	126. 83	149. 41	108. 82	141. 30	110. 69	109. 46	114. 19

**F3**	16	130. 77	121. 17	131. 11	133. 99	143. 29	131. 57	146. 84	126. 35	152. 52	129. 60	139. 15	136. 29

**C3**	16	143. 39	103. 03	161. 99	106. 40	184. 32	125. 74	199. 06	**122. 00**	163. 24	119. 82	131. 87	91. 23

**P3**	16	125. 22	101. 65	139. 11	129. 07	127. 10	129. 55	135. 28	129. 60	112. 85	123. 79	107. 19	110. 95

**Pz**	16	122. 58	102. 78	118. 77	117. 04	138. 91	122. 57	140. 81	134. 61	122. 99	122. 09	116. 98	120. 23

**P4**	16	169. 23	90. 46	161. 94	123. 27	146. 17	126. 59	149. 79	**123. 37**	132. 53	112. 93	124. 39	106. 21

**C4**	16	108. 49	91. 85	139. 05	111. 22	154. 92	144. 81	179. 28	125. 38	132. 69	129. 46	137. 85	113. 68

**F4**	16	117. 70	138. 61	129. 74	130. 22	158. 47	**118. 51**	154. 69	109. 41	162. 97	130. 81	134. 72	114. 71

**F7**	16	122. 49	180. 62	154. 49	152. 29	146. 71	147. 22	131. 11	147. 22	137. 28	132. 59	141. 24	145. 14

**T3**	16	155. 39	151. 76	218. 06	137. 20	210. 65	**141. 20**	212. 31	**138. 22**	143. 21	127. 92	128. 86	91. 49

**T5**	16	132. 15	144. 40	153. 07	134. 47	153. 93	153. 34	147. 75	136. 95	123. 87	129. 40	109. 77	115. 14

**O1**	16	136. 22	108. 16	137. 12	124. 71	166. 38	118. 52	171. 29	**115. 74**	170. 13	122. 99	140. 16	95. 78

**O2**	16	118. 31	101. 77	154. 50	121. 48	162. 00	114. 22	152. 19	118. 28	143. 77	116. 68	131. 18	107. 77

**T6**	16	175. 11	147. 15	156. 15	140. 95	154. 38	145. 39	138. 18	130. 82	119. 30	116. 71	106. 77	109. 75

**T4**	16	156. 82	167. 62	155. 88	142. 24	173. 66	157. 10	142. 40	148. 35	140. 33	134. 77	137. 56	111. 50

**F8**	16	167. 63	130. 59	138. 15	144. 36	141. 56	145. 84	137. 09	115. 38	139. 92	117. 06	132. 92	117. 37

**Me**	16	130. 77	108. 16	139. 11	129. 07	154. 38	131. 57	149. 41	126. 35	139. 92	123. 79	131. 18	113. 68

Relative power values (% of pre-drug) for each electrode position 4 h after administration of drug or placebo during relaxation under the recording condition of eyes open 4 h after intake of placebo or verum. Statistically significant different values in comparison to placebo (p = 0. 021) are printed **fat**. E = Electrode; Me = Median; n = number of subjects.

### Analysis of Single Brain Regions Under Mental Load

**Figure 2:**
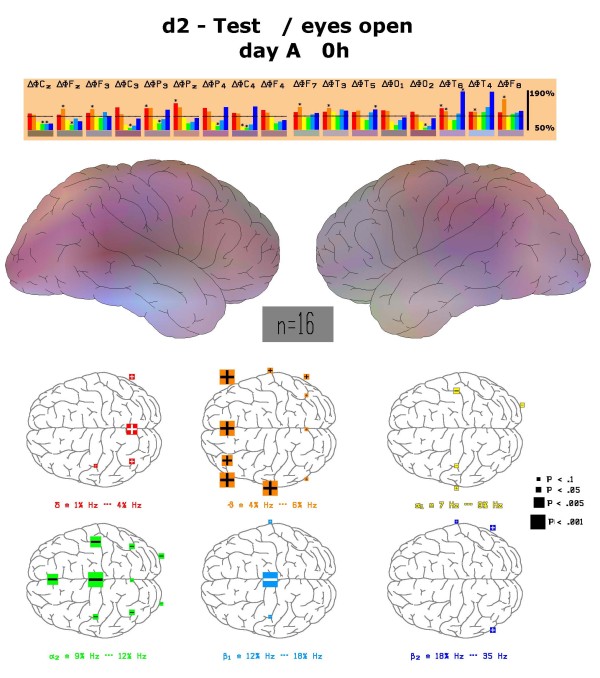
**Physiological changes of spectral frequency content during d2 test**. Depiction of frequency changes *at all electrode positions *during the performance of the d2 test in comparison to the relaxed eyes-open state at the first visit (day A). Colour vision map with interpolated data is constructed as described under methods. Statistically significant changes between these two states are marked by an asterisk and depicted with respect to position in the map. Left side of figure corresponds to frontal brain region.

Different mental tasks produce changes of electric brain activity within several anatomically defined brain regions. According to earlier experience a concentration test induces spectral frequency changes which can be differentiated from those in the presence of memory testing or performance of mathematical tasks. If one compares the spectral changes during the performance of the d2 test with the relaxed eyes open situation, brain regions react in a different reproducible manner also in this study. Fronto-temporal electrode positions show increases of theta power whereas in other regions alpha1, alpha2 and central beta1 power provide considerably lower values (Figure 2). These changes in comparison to the eyes open situation are statistically significant with p < 0. 05 for particular electrode positions (labelled by stars in the upper part of Figure 2). Documentation of these changes within a brain map containing all frequencies by additive colour mixture (for details s. methods) reveals more increases of theta power within left frontal cortex which leads to orange colour dominance on this side (middle part of Figure 2). Details of the statistical evaluation are given in the lower part of Figure 2 corresponding to the map. Highest significances for frequency changes during performance of the d2 test can be documented for increased fronto-temporal theta power and decreased alpha2 and beta1 power in other regions. Statistically significant decreases of alpha2 power were observed for the electrode positions Cz, C3, C4, Fz, P3, P4 and O2.

**Figure 3:**
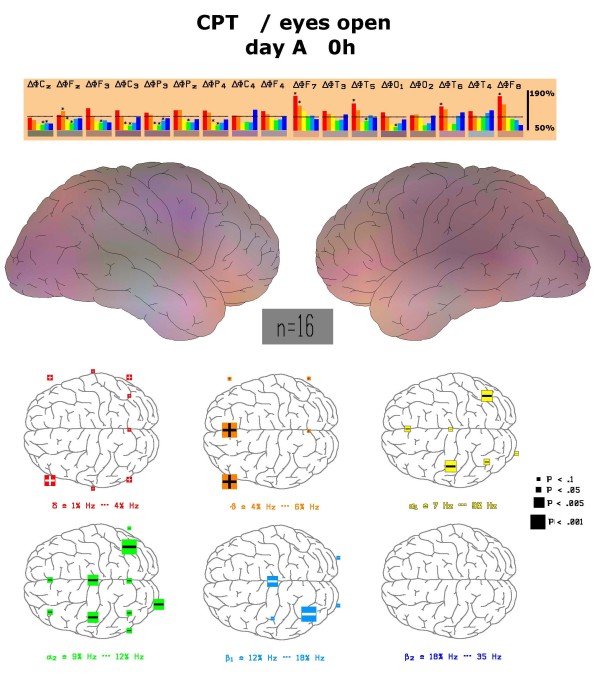
**Physiological changes of spectral frequency content during CPT**. Depiction of frequency changes *at all electrode positions *during the performance of the d2 test in comparison to the relaxed eyes-open state at the first visit (day A) before drug administration. Colour vision map with interpolated data is constructed as described under methods. Statistically significant changes between these two states are marked by an asterisk star and depicted with respect to position in the map. Left side of figure corresponds to frontal brain region.

A similar picture arises during the performance of the concentration performance test (CPT), where mathematical calculations have to be done. Large increases of left and middle frontal theta power are accompanied by massive decreases mainly in centro-parietal alpha2 spectral power involving also some areas with decrease in beta1 power. These changes in comparison to the relaxed eyes open status are also statistically highly significant (labelled by stars in Figure 3). Increases of left frontal theta power are also recognized in the map (middle part of Figure 3). Statistically significant decreases of alpha2 power in other regions were observed for the electrode positions Cz, C3, Fz, P3, Pz, P4 and O1.

**Figure 4:**
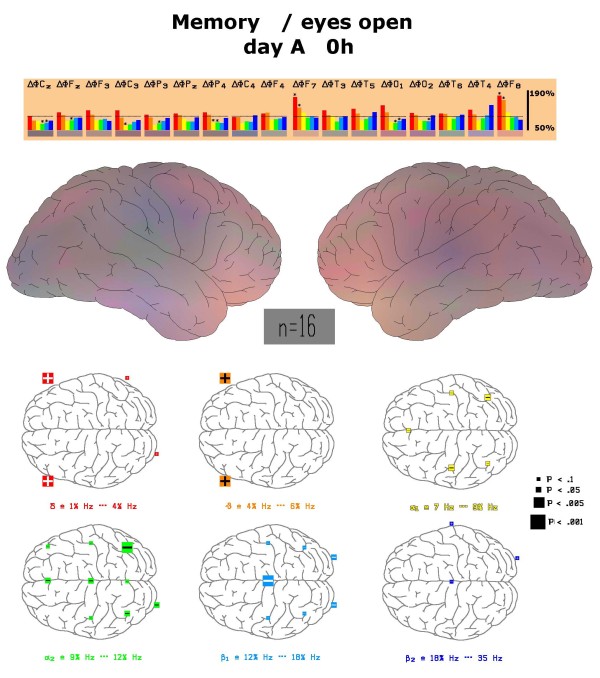
**Physiological changes of spectral frequency content during memory test**. Depiction of frequency changes *at all electrode positions *during the performance of the d2 test in comparison to the relaxed eyes-open state at the first visit (day A). Colour vision map with interpolated data is constructed as described under methods. Statistically significant changes between these two states are marked by an asterisk and depicted with respect to position in the map. Left side of figure corresponds to frontal brain region.

During the performance of the memory test, where a row of 8 combined letters and numbers have to be memorized within 4 seconds, also left and right frontal delta and theta power increase in a highly significant manner (labelled by stars in the upper part of Figure 4) which also leads to dominant red-yellow colour increases in the brain map (middle part of Figure 4). Details of the statistically significant differences in comparison to the relaxed status are documented in the lower part of Figure 4. Statistically significant decreases of alpha2 power were observed for the electrode positions Cz, Fz, P3, P4 and O1.

Thus, all three mental loads lead to somewhat different changes of frequency patterns in comparison to the relaxed state but have in common a fronto-temporal increase in delta and/or theta power with significant decreases of alpha and beta1 power in other brain regions.

### Analysis of Drug Effects in the Presence of Various Mental Loads

No significant differences between verum and placebo were seen with regard to fronto-temporal delta/theta power increases. In order to better interpret the effects of the drug on electric spectral power, medium power of those electrode positions involved in the statistically significant (at least by p < 0. 05) decrease of alpha2 power during mental work were unified to give one parameter set. They represent a brain region heavily involved in the process of a particular mental task performance.

**Figure 5:**
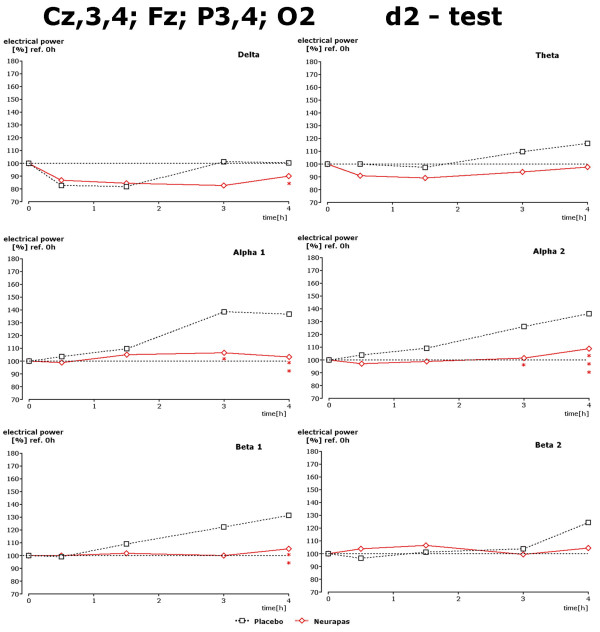
**Drug-induced changes of spectral frequency content during d2 test**. Time dependence of spectral frequency changes during performance of the d2 test in the presence of placebo or verum. Choice of electrode positions in concordance with statistically significant changes observed during this test condition (s. Figure 3). * corresponds to p < 0. 12; ** corresponds to p < 0. 076; *** corresponds to p < 0. 02.

**Figure 6:**
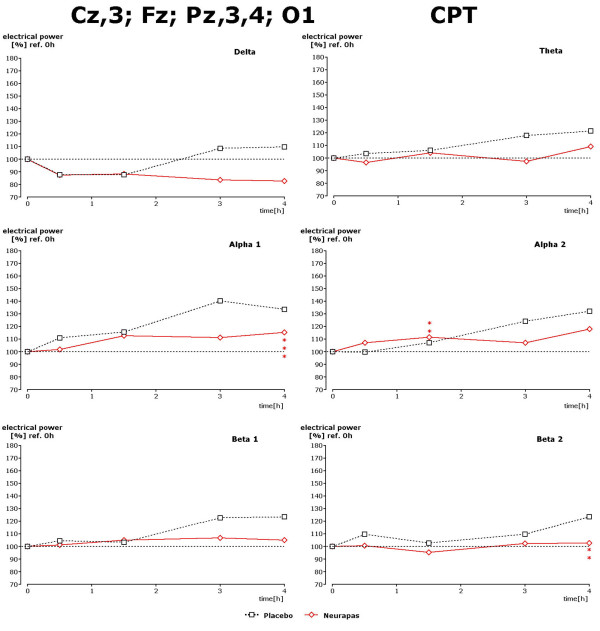
**Drug-induced changes of spectral frequency content during CPT**. Time dependence of spectral frequency changes during performance of the CPT in the presence of placebo or verum. Choice of electrode positions in concordance with statistically significant changes observed during this test condition (s. Figure 4). * corresponds to p < 0. 12; ** corresponds to p < 0. 076; *** corresponds to p < 0. 02.

**Figure 7:**
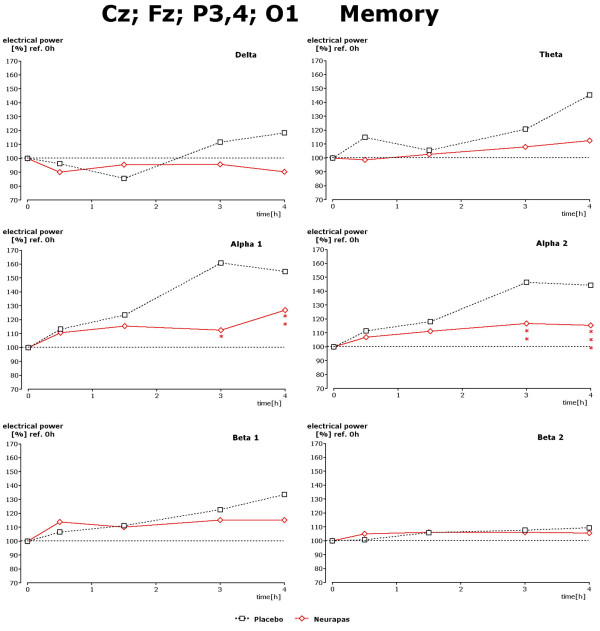
**Drug-induced changes of spectral frequency content during memory test**. Time dependence of spectral frequency changes during performance of the memory test in the presence of placebo or verum. Choice of electrode positions in concordance with statistically significant changes observed during this test condition (s. Figure 5). * corresponds to p < 0. 12; ** corresponds to p < 0. 076; *** corresponds to p < 0. 02.

Recording under the three different mental performance conditions succeeded in uncovering drug-induced changes within the alpha and beta1 spectral frequencies during the 3^rd ^and 4^th ^hour after drug administration. Results for the d2 test are given in Figure 5. Under this recording condition statistically significant lower power values of alpha1, alpha2 and beta1 power were observed 3 and/or 4 hours after administration of verum in comparison to placebo. Regarding the data during performance of the CPT again statistically significantly lower values were obtained during the 4^th ^hour after administration for theta, alpha1 and beta1 spectral power (Figure 6). During the 3^rd ^and 4^th ^hour performance of the memory test led to significantly diminished values of alpha1 and alpha2 spectral power in comparison to placebo, too (Figure 7).

Thus, during all mental tasks alpha and beta1 power was statistically significantly lower in the presence of verum in comparison to placebo. Analysis of the psychometric results revealed either no difference (Table 4) or a trend of better performance for the CPT at 3 and 4 hours after administration (Table 5), which, however, was not statistically significant, or no difference during the memory test (Table 6).

**Table 4:** Result of psychometric procedures d2-test

d2-test results			
		Placebo	NEURAPAS^®^
**0 h**	**Mean**	**12,32**	**12,14**
	SD	4,02	3,06
	SEM	1,07	0,80
**0,5 h**	**Mean**	**13,67**	**13,09**
	SD	3,82	3,27
	SEM	1,02	0,84
**1,5 h**	**Mean**	**13,62**	**13,71**
	SD	4,22	3,89
	SEM	1,12	0,91
**3 h**	**Mean**	**13,35**	**13,19**
	SD	3,95	3,27
	SEM	1,05	0,84
**4 h**	**Mean**	**13,59**	**13,53**
	SD	3,77	3,13
	SEM	1,01	0,82

Listing of psychometric results for three different mental tests:

**Table 5:** Result of psychometric procedures CPT

CPT results			
		Placebo	NEURAPAS^®^
**0 h**	**Mean**	**6,57**	**5,59**
	SD	6,17	5,95
	SEM	1,61	1,59
**0,5 h**	**Mean**	**5,32**	**5,91**
	SD	4,57	4,94
	SEM	1,20	1,25
**1,5 h**	**Mean**	**6,55**	**5,85**
	SD	5,98	4,93
	SEM	1,58	1,29
**3 h**	**Mean**	**5,93**	**6,97**
	SD	5,33	5,83
	SEM	1,41	1,52
**4 h**	**Mean**	**6,87**	**7,86**
	SD	7,30	5,76
	SEM	1,93	1,52

Listing of psychometric results for three different mental tests:

**Table 6:** Result of psychometric procedures Memory

Memory results			
		Placebo	NEURAPAS^®^
**0 h**	**Mean**	**10,77**	**11,37**
	SD	3,98	3,64
	SEM	1,06	0,92
**0,5 h**	**Mean**	**10,95**	**11,38**
	SD	4,10	3,60
	SEM	1,10	0,96
**1,5 h**	**Mean**	**10,68**	**11,29**
	SD	4,46	3,13
	SEM	1,19	0,80
**3 h**	**Mean**	**11,11**	**12,12**
	SD	4,38	3,75
	SEM	1,07	1,00
**4 h**	**Mean**	**11,51**	**11,95**
	SD	3,74	3,65
	SEM	0,99	0,92

Listing of psychometric results for three different mental tests:

## Discussion

**Figure 8:**
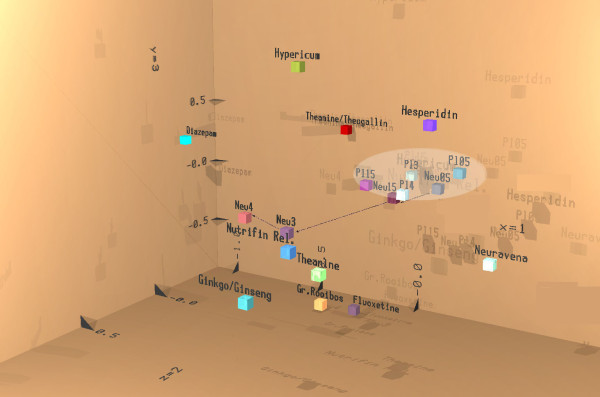
**Discriminant Analysis of EEG source density data**. Depiction of the result of linear discriminant analysis of EEG source density data for all four recording periods after administration of verum and placebo. Result from the first three discriminant functions is displayed by means of space coordinates x, y and z. Values of the 4^th ^to 6^th ^linear discriminant functions respectively determine the amount of red, green, and blue in additive colour mixture analogous to the RGB mode of TV.

Since it is quite difficult to evaluate the data from 17 electrode positions in the presence of 6 frequency ranges (a total of 102 parameters) the mathematical tool of discriminant analysis was used to describe the action of the active drug in comparison to placebo for the recording condition eyes open. This method allows time-dependent evaluation of all changes with respect to the pre-drug condition. As can be seen in Figure 8 all placebo values (labelled PL05 to PL4)-representing circadian rhythm dependent changes-group together with the two first recordings from active drug at 0. 5 to 1. 5 hours after administration (labelled Neu05 and Neu15). Depiction of the result of the effect of verum at 3 and 4 hours after administration in comparison to the effect of other synthetic reference drugs or plant-derived preparations tested earlier revealed clear effects for the active drug (labelled Neu3 and Neu4 in Figure 8). Closest neighbours in space (representing the result of the first to third discriminant function) but with a different colour due to the result of the fourth to sixth discriminant function are Nutrifin-relax and L-Theanine. Another neighbour not too far away with the same colour is fluoxetine.

This kind of analysis of the experimental series revealed that the preparation NEURAPAS^® ^balance modulated electric brain activity already after a single acute administration in a statistically significant manner in comparison to placebo. Three and four hours after the administration of verum, basic activity has changed to a considerable degree in comparison to placebo according to this analysis. The pattern of changes approached that seen after reference drugs or preparations like Nutrifin-relax, a plant-derived preparation marketed to cope with stress [9]. Furthermore, fluoxetine, a chemical reference drug with clinically proven anti-depressive effects [17] is projected within the documentation of the result of discriminant analysis more close than to a preparation of St. John's wort tested earlier under identical conditions (results not shown). Comparison with a previous study shows that the projection of Neurapas^® ^balance is close to that of ginkgo/ginseng. This vicinity of the projection of gingko/ginseng effects on electric activity of the brain suggests beneficial effects [18], since clinically significant improvement in memory loss, concentration, fatigue, anxiety and depressed mood associated with ginkgo/ginseng has been reported in the literature [19]. This suggests that NEURAPAS^® ^balance might unify anti-depressive and relaxing features as claimed by the design of the combination according to known effects of single extracts from St. John's wort, valerian, and passion flower (see reference under "background ").

Spectral power of frequencies, which predominantly were attenuated during performance of mental work in more centro-parietal areas of the brain, remain low 3 and 4 hours after administration of the active preparation in comparison to placebo (which led to an increase of spectral power rather than the decrease observed in the pre-drug condition!). This suggests that mental processing ability is relatively preserved 3-4 hours after administration of NEURAPAS^® ^balance compared to the placebo condition, where increases of alpha and beta spectral power were observed presumably due to circadian rhythms and/or fatigue. Even if there was no difference in psychometric results, some higher values were noted with respect to the concentration performance test (CPT), where highest performance was recorded during the 3^rd ^and 4^th ^hour after administration, perhaps related to the relative constancy of EEG spectra.

For St. John's wort maximum effects after single dose administration have been observed between 4 and 6 hours after administration [20]. This is in line with the current results, where no effects could be recognized before the 3rd hour after administration. Better cognitive functioning in the presence of one of the ingredients (St. John's wort) has been claimed on the basis of EEG recording by [21]. But, due to the combination of three plant-derived extracts the effects cannot be attributed to one of them. However, it is important to note, that at least no interference with cognitive performance could be seen as reported repeatedly during the use of other synthetic preparations like benzodiazepines as anti-anxiety drugs or serotonin-reuptake inhibitors for treatment of depression [1]. With respect to the rationale of combining the three extracts a more potent neuroactivity of the triple combination compared to Hypericum alone and the additive effects of Passiflora and Valeriana suggested a synergy between constituents of these herbal extracts as derived from in vitro experiments with multielectrode neurochips [22]. This study indicated that the ingredients of NEURAPAS^® ^balance act on GABA and serotonin receptors. Involvement of the GABAergic transmission has also been claimed for one of the constituents of the triple combination (Passiflora incarnate) by in vivo trials by [23] as well as in vitro experiments [24].

EEG frequencies reflect the activity of particular neurotransmitter activities as discovered earlier in preclinical studies using recording from depth electrodes in freely moving rats. From these earlier preclinical experiments is became clear that alpha1 waves seem to be related to serotonergic transmission [25] and that alpha2 waves seem to be related to the activity of dopamine [26]. Based on these preclinical results one might interpret the data obtained with respect to NEURAPAS^® ^balance in a sense that ingredients contained within this extract combination activate serotonergic and dopaminergic transmission. These two transmitters are well known to be involved in depression and anxiety. Synthetic drugs acting at receptors of these two neurotransmitters are prescribed first line to treat these psychiatric conditions, especially serotonin-reuptake inhibitors for the treatment of depression. Some decreased values of alpha1 and alpha2 waves during the recording condition "eyes open" are therefore in line with the view that NEURAPAS^® ^balance activates these transmitter systems and initiates its action already after the first intake.

Finally, the question of combining different extracts must be tackled. According to literature changes of the EEG in the presence of for example valerian roots extract could not be shown, even at dosages as high as 300 or 600 mg [27]. Also with respect to St. John's wort dosages as high as 900 mg had to be given in order to see changes of electrical activity [20]. Positive results with an extract from passion-flower were obtained with a dose of 425 mg native extract (to be published). Thus, the relatively low amounts of the single extracts combined within NEURAPAS^® ^balance speak in favour of not only additive but potentiating effects of single ingredients from St. John's wort herb, valerian root, and passion flower herb in order to produce the electric profile of effects on the human brain as described above.

## Conclusions

Administration of NEURAPAS^® ^balance resulted in a prevention of circadian EEG changes which normally consist in increases of spectral power in all six frequency ranges. Under the condition of mental work this attenuation became statistically significant for alpha and partially beta waves at 3 and 4 hours after intake. No cognitive impairment could be shown during the recording period according to analysis of psychometric testing. Linear discriminant analysis showed that the effects of NEURAPAS^® ^balance are more comparable to the effects of Fluoxetine (a synthetic antidepressant drug) than to those in the presence of St. John's wort extract without producing side effects.

## Competing interests

The authors declare that they have no competing interests.

## Authors' contributions

WD provided the electrophysiological technology, supervised the performance of the experiments, gave interpretation of the results and wrote the manuscript. KK performed the medical examinations and evaluated unexpected events. GW initiated the study and made major contributions to the design. She also provided important information on the pharmacology of single as well as of the combined constituents of the preparation.

The authors read and approved the manuscript.

## Pre-publication history

The pre-publication history for this paper can be accessed here:

http://www.biomedcentral.com/1471-244X/11/123/prepub
